# Editorial: Physical activity and fitness for the prevention and management of bone diseases

**DOI:** 10.3389/fphys.2023.1185201

**Published:** 2023-04-12

**Authors:** Esther Ubago-Guisado, Jaak Jürimäe, Luis Gracia-Marco

**Affiliations:** ^1^ Escuela Andaluza de Salud Pública (EASP), Granada, Spain; ^2^ Instituto de Investigación Biosanitaria, ibs.Granada, Granada, Spain; ^3^ Institute of Sports Sciences and Physiotherapy, University of Tartu, Tartu, Estonia; ^4^ Department of Physical Education and Sports, Faculty of Sport Sciences, Sport and Health University Research Institute (iMUDS), University of Granada, Granada, Spain; ^5^ Centro de Investigación Biomédica en Red Fisiopatología de la Obesidad y Nutrición (CIBERobn), Instituto de Salud Carlos III, Madrid, Spain

**Keywords:** exercise, bone mineral density, non-communicable diseases, children, adults, mice, DXA, pQCT

Osteoporosis, the most common bone disease, is a major non-communicable disease (NCD) and a public health problem. Approximately 50% of women and 20% of men will suffer at least one osteoporosis fracture above the age of 50 years (Holroyd et al., 2008). Physical inactivity and poor fitness are known as important factors behind the rise in NCDs and have been linked poor bone health and bone metabolism (Gil-Cosano et al., 2020; Ubago-Guisado et al., 2020; Jürimäe et al., 2021). There is a lack of proper exercise intervention studies specifically designed for improving bone outcomes at clinical sites at various life stages and under certain conditions, which is crucial to underline the importance of physical activity and fitness both on bone accrual and bone preservation. Research have predominantly used Dual-energy X-ray Absorptiometry (DXA) scans to quantify bone mass but data from 3-dimensional devices is scarce and may provide with additional cortical and trabecular bone parameters to better understand bone adaptations to exercise. This Research Topic of Frontiers in Physiology, “Physical activity and fitness for the prevention and management of bone diseases” contains 11 publications (9 original manuscripts and 2 systematic reviews and meta-analyses) investigating the contribution of exercise-induced mechanical stimulation on relevant bone outcomes in a variety of populations.

Two timely systematic reviews and meta-analyses were included in this Research Topic. Marmol-Perez et al. showed that the reduced number of exercise interventions performed to date (*n* = 8 studies) were inappropriate and therefore, ineffective to obtain any beneficial effect on bone health in children and adolescents with cancer during and after oncological treatment. Wu et al. assessed the effectiveness of Tai Chi on adult patients with rheumatoid arthritis (*n* = 9 studies) and observed that Tai Chi was a safe method in this population yet it did not improve pain or physical function outcomes.

Four original studies were published in young populations. Wang et al. performed a randomized controlled trial to investigate the effects of a 14-week cheerleading programme on areal bone mineral density (aBMD) outcomes (measured by DXA) and advanced glycosylation end products in older adolescents (*n* = 46). Their findings support the use of cheerleading as a non-pharmacological intervention to improve aBMD (and physical fitness) by reducing advanced glycosylation end products. Kurgan et al. performed a randomized controlled trial to examine the influence of a 12-week (combined) exercise training and nutritional counselling (dairy intake) intervention on bone remodelling and metabolism in adolescent females with overweight/obesity (*n* = 30). In this study, the authors found that the intervention blunted the increase in sclerostin and augmented the increase in osteoprotegerin (OPG): receptor activator of nuclear factor kappa B Ligand (RANKL), following acute exercise. Interestingly, dairy product consumption did not further influence these responses. Mello et al. investigated the accuracy of different physical fitness tests for the screening of low aBMD in children aged 6–11 years (*n* = 160). In the same sample, Mello et al. also examined the associations between the performance in commonly used fitness tests in young populations with the aBMD obtained in different regions, observing site- and- test specific associations.

Three original studies were published in adult populations. Two of them, Fernandez et al. and Buchanan et al. used whole-body vibration training to stimulate bone. Fernandez et al. conducted a 12-month non-randomized clinical trial to evaluate the effects of high-frequency and combined amplitude stimuli whole-body vibration on bone parameters and bone metabolism markers in physically inactive postmenopausal women (*n* = 255) with 10-year major osteoporotic fracture risk. In their study, DXA was used to measure aBMD and high-resolution peripheral quantitative computed tomography (HR-pQCT) to measure bone microarchitecture. Despite the protocol was well tolerated, the study failed to detect a significantly change in any of the bone outcomes nor an amelioration was observed. Buchanan et al. performed a randomized crossover design study with 10 postmenopausal women to compare the responses of selected circulating microRNAs to a bout of resistance exercise and a bout of whole-body vibration and, its association with a bone resorption marker (tartrate-resistant acid phosphatase 5b, TRAP5b) The authors found that whole-body vibration altered circulating miR-21-5p expression and that increases in TRAP5b were associated with greater downregulation of this expression. Stunes et al. used a cross-over study design to evaluate the acute effects of two exercise sessions (resistance training and high-intensity interval training) on bone turnover markers in young adults of both sexes (*n* = 39) and elderly men (*n* = 14). The authors observed no differences in the response of most bone turnover markers between the sessions. However, the altered response of bone turnover markers was generally blunted after 24 h.

Two original studies were published in mice. Massing et al. in their randomized controlled trial (six experimental groups) compared the effects of various 14-week treadmill exercise programs on the loss of cortical and trabecular structures in postmenopausal ovariectomized C57BL/6J mice (*n* = 42 female mice). Micro-computed tomography (μCT) was used to measure bone structures and data on bone turnover markers was also obtained. Ovariectomy induced bone loss, however, none of the treadmill exercise programs was beneficial in preventing the loss of bone mass, challenging the idea that just treadmill training is suitable to stop ovariectomy-related bone loss. Finally, Huesa et al. investigated the effects of 3–7 weeks of moderate exercise (forced wheel walking) on early joint pathology in the mouse destabilization of the medial meniscus model (*n* = 42 male mice). Histology and μCT were used to assess the joints. Findings showed the protective effect of exercise against cartilage damage after 7 weeks of exercise, and a temporary protection against osteosclerosis after 3 weeks of exercise.

The present Research Topic provides a short summary of the progress on the topic of exercise and bone disease which will be useful from a clinical and public health perspective. It also highlights the current limitations and the necessity of more powerful study designs to further advance in the knowledge. Bone adaptations to exercise are greater during growth due to the bone cellular activity and therefore, preventative measures are highly recommended to build strong bones and reduce the odds of suffering bone diseases later in life. Researchers, when possible, must consider performing randomized controlled trials and avoid short interventions since the remodelling cycle may take at least 4 months for cortical bone and 7–8 months for trabecular bone (Agerbaek et al., 1991). In this sense, it is highly recommended implementing behavioural change techniques that increase motivation and therefore, adherence to the programmes. Whenever possible, the combination of DXA and QCT scans with bone turnover markers is also recommended to obtain a wider picture of bone adaptations.
